# Preoperative partial breast reirradiation and repeat breast-conserving surgery in patients with recurrent breast cancer: the prospective single-arm REPEAT trial – a study protocol

**DOI:** 10.1136/bmjopen-2024-096510

**Published:** 2025-07-18

**Authors:** Yasmin Civil, Lisca Wurfbain, Lysanne Jonker, Maurice van der Sangen, Arlene Oei, Katya Duvivier, Nina Bijker, Philip Meijnen, Zdenko van Kesteren, Miguel Palacios, Ellis Barbé, Willemien Menke-van der Houven van Oordt, Gwen Diepenhorst, Victor Thijssen, Berend Slotman, Joost Verhoeff, Robert-Jan Schipper, Desirée van den Bongard

**Affiliations:** 1 Department of Radiation Oncology, Amsterdam UMC Location VUmc, Amsterdam, Noord-Holland, The Netherlands; 2 Cancer Treatment and Quality of Life, Cancer Center Amsterdam, Amsterdam, The Netherlands; 3 Department of Radiation Oncology, Catharina Hospital, Eindhoven, Noord-Brabant, The Netherlands; 4 Laboratory for Experimental Oncology and Radiobiology (LEXOR), Center for Experimental Molecular Medicine (CEMM), Amsterdam UMC Location VUmc, Amsterdam, Noord-Holland, The Netherlands; 5 Cancer Biology and Immunology, Cancer Center Amsterdam, Amsterdam, The Netherlands; 6 Department of Radiology, Amsterdam UMC Location VUmc, Amsterdam, Noord-Holland, The Netherlands; 7 Department of Pathology, Amsterdam UMC Location VUmc, Amsterdam, Noord-Holland, The Netherlands; 8 Department of Medical Oncology, Amsterdam UMC Location VUmc, Amsterdam, Noord-Holland, The Netherlands; 9 Department of Surgery, Amsterdam UMC Location VUmc, Amsterdam, Noord-Holland, The Netherlands; 10 Department of Surgery, Flevo Hospital, Almere, Flevoland, The Netherlands; 11 Department of Surgery, Catharina Hospital, Eindhoven, Noord-Brabant, The Netherlands

**Keywords:** Breast tumours, RADIOTHERAPY, Quality of Life, Radiation oncology, Toxicity

## Abstract

**Introduction:**

Over the past decades, interest in second breast-conserving therapy (BCT) has increased due to, among others, advances in radiotherapy techniques. Preoperative partial breast irradiation (PBI) is an experimental treatment for patients with low-risk primary breast cancer. This approach can downstage the tumour and may possibly reduce toxicity and improve cosmetic outcomes compared with postoperative radiotherapy. This study aims to evaluate the feasibility of single-dose preoperative PBI and second breast-conserving surgery (BCS) for patients with an ipsilateral recurrent breast event (IRBE) after previous BCT.

**Methods and analysis:**

The REPEAT trial is a multicentre, prospective, single-arm trial investigating ablative single-dose preoperative PBI in patients with an IRBE. Eligible patients are ≥50 years, have a unifocal non-lobular invasive breast cancer ≤2 cm, Bloom-Richardson grade 1 or 2, oestrogen receptor-positive, human epidermal growth factor receptor 2-negative and clinically negative axillary lymph nodes. The study plans to enrol 25 patients. Radiotherapy planning will involve the use of CT and MRI in the treatment position. Single-dose PBI of 20 Gy to the tumour and 15 Gy to the surrounding 2 cm of breast tissue will be delivered using a conventional or MR-guided linear accelerator. Tumour response will be monitored preoperatively using MRI and liquid biopsies to identify biomarkers for evaluating radiosensitivity. BCS will be performed 3 (±one) weeks post PBI. The primary endpoint is the incidence of grade 2 or higher treatment-associated acute toxicity within 90 days. Secondary endpoints include the evaluation of acute (grade 1) and late toxicity, radiologic and pathologic response, mastectomy rate, patient-reported outcomes, cosmetic outcome, local, regional and distant recurrence rates, survival outcome and biomarkers in liquid biopsies and tumour tissue. Patients will be followed up to 5 years after PBI.

**Ethics and dissemination:**

Ethical approval from the Medical Research Ethics Committee of the Amsterdam UMC has been obtained (NL85983.018.24). The results will be disseminated via peer-reviewed academic journal and presentation at conferences. In addition, summaries will be shared with the participating patients.

**Trial registration number:**

The trial was registered prospectively on October 11^th^ 2024 at clinicaltrials.gov (NCT06640881).

**STRENGTHS AND LIMITATIONS OF THIS STUDY**

- This study offers a comprehensive set of outcome measures, providing valuable insights into the safety of preoperative single-dose partial breast irradiation following previous breast-conserving therapy. It also assessed quality of life, cosmetic outcomes and radiosensitivity biomarkers.
- A notable strength is the study’s design, which allows for easy implementation into clinical practice by conducting axillary surgery as per recommendations of a multidisciplinary tumour board, given the debated value of the sentinel node procedure as a staging tool.
- We evaluate late toxicity, quality of life, cosmetic outcome and oncological outcomes over a 5-year follow-up.
- A limitation is the short interval between radiotherapy and surgery, which ensures oncological safety but may not allow sufficient time to observe a radiotherapy-induced pathological tumour response.

## Background

 The majority of patients with early-stage breast cancer are treated with breast-conserving therapy (BCT), consisting of breast-conserving surgery (BCS) followed by whole breast irradiation (WBI) or partial breast irradiation (PBI).^1^,^5^ The 5-year local recurrence rate after BCT is low, 0.5% per year.^6^ However, due to improved breast-cancer survival, the absolute number of patients with an ipsilateral recurrent breast event (IRBE) is increasing.^7^ Current standard for care of an IRBE is salvage mastectomy after previous BCT.^8^

Over the last decades, the interest in second-BCT in patients with a low-risk ipsilateral breast cancer or ductal carcinoma in situ (DCIS) event has increased.^9 10^ These low-risk characteristics are age ≥50 years, invasive tumour size ≤2 cm or DCIS <2.5 cm, Bloom-Richardson grade 1–2, oestrogen (ER)-positive, human epidermal growth factor receptor 2 (HER2)-negative, node negative breast cancer, with a time interval after primary breast cancer ≥2 years.^9 10^ A recent systematic review of single-arm retrospective cohort studies in patients with an IRBE after initial BCT showed weighted estimates for 5-year overall survival (OS) for repeat BCS alone and repeat BCS and postoperative reirradiation of 77.0% (95% CI 76.1% to 78.7%) and 87.3% (95% CI 86.9% to 87.7%), respectively.^9^ 5-year local control was 76.0% (95% CI 74.5% to 76.9%) for repeat BCS and 88.6% (95% CI 87.9% to 89.2%) for repeat BCS followed by postoperative reirradiation.^9^ Two studies on BCS with partial breast reirradiation using external beam radiotherapy (RT) and brachytherapy reported 5-year OS rates of 95% (95% CI 85% to 98%) and 89% (95% CI 86% to 93%), respectively.^6 11^ The 5-year cumulative incidence of IRBE was 5% (95% CI 1% to 13%) and 4% (95% CI 2% to 6%), respectively. Second BCT might not be the preferred treatment for patients who already had poor cosmetic outcome after initial BCT, as reirradiation could worsen cosmetic outcome due to the risk of additional breast fibrosis.

There is increasing interest in preoperative RT in patients with low-risk primary breast cancer, since target definition is more precise and the irradiated volume is smaller compared with postoperative RT.^12 13^ Consequently, preoperative PBI could reduce RT-induced toxicity and improve quality of life (QoL). A smaller volume also enables a higher radiation dose per fraction which results in a reduced number of fractions and overall treatment time. A recent systematic review showed that grade 1 acute skin toxicity (0–34%), grade 1 seroma and grade 1 breast fibrosis (46–100%) were the most frequently reported toxicities.^12^ Cosmetic outcome was good to excellent in 78% to 100% of the patients. After a maximum median follow-up of 5 years, local recurrence rates were 0 to 3% and OS 97–100%.^12^ It is expected that reirradiation using preoperative PBI will lead to smaller irradiated volumes equal to preoperative PBI in primary low-risk breast cancer. This could result in a limited dose to the surrounding organs at risk and could lead to less toxicity and better cosmesis compared with postoperative PBI after second BCS. In addition, the breast tissue exposed to the highest RT dose is excised during surgery, reducing the risk of RT-associated toxicity. To our knowledge, there are no data on preoperative reirradiation in patients with an IRBE after previous BCT.

The REPEAT trial aims to assess the feasibility of single-dose preoperative PBI and second BCS for an IRBE after previous BCT based on grade 2 or higher acute toxicity. Further response assessment modalities will be evaluated to optimise response assessment with radiological evaluation using breast MRI and the analysis of biomarkers in blood and tumour tissue. In addition, acute and late toxicity, pathological response, patient-reported outcome measures (PROMs), cosmetic results and oncological outcomes will be evaluated.

## Methods/design

### Study design

The REPEAT trial is a Dutch multicentre, phase II, single-arm prospective study at the Amsterdam University Medical Center (UMC) and Catharina Hospital Eindhoven in the Netherlands. The trial started in both centres in January 2025 and the estimated end date is December 2032. Eligible patients will be treated with ablative single-dose PBI followed by BCS after 3 (±one) weeks. At baseline (prior to RT) and prior to BCS, toxicity, PROMs and cosmetic outcome will be assessed. Additionally, a preoperative MRI will be performed 3 (±one) weeks following RT to evaluate the acute tumour response. The primary objective is to assess the feasibility of single-dose preoperative PBI and second BCS for an IRBE after previous BCT based on acute toxicity. The secondary objectives are to assess treatment-induced toxicity, radiological and pathological response, mastectomy rate, PROMs, cosmetic results, patient comfort during RT treatment delivery, oncological outcomes and RT-associated immune response markers (CD4, CD8 and FOXP3 expressing T cells) in blood and tumour tissue. All tumour tissue and blood samples of patients will be preserved at the Amsterdam UMC Central Biobank, location Vrije Universiteit Medical Center. This study design is based on the ABLATIVE study in patients with primary low-risk breast cancer, but with a shorter interval between RT and BCS (NCT05350722).^14 15^

### Study population

The study population will consist of women who are 50 years or older with a low-risk IRBE after previous BCT. The definition of low-risk IRBE is based on the ASTRO suitable criteria for PBI.^1^ Patients are eligible for study participation in case of: tumour size ≤2 cm on MRI, unifocal non-lobular invasive carcinoma, Bloom-Richardson grade 1 or 2, ER-positive and HER2-negative, clinical node negative on 18F-fluorodeoxyglucose positron emission tomography computed tomography (FDG PET-CT)/ultrasound/MRI, no distant metastases and no or mild late toxicity (no grade 2 or higher) from previous BCT. The inclusion and exclusion criteria are presented in table 1.

**Table 1:** Inclusion and exclusion criteria

Inclusion criteria	Exclusion criteria
WHO performance status 0–2	Ipsilateral invasive breast cancer event <2 years after first breast-conserving therapy for breast cancer
Women ≥50 years with an ipsilateral invasive recurrent breast cancer event after previous breast-conserving surgery and postoperative whole breast irradiation	Other malignancy within 5 years before ipsilateral breast recurrence diagnosis. For carcinoma in situ, no specific time span to ipsilateral breast recurrence diagnosis is required for inclusion
Tumour size ≤2 cm and unifocal on MRI	Known breast cancer mutation gene carrier
Tumour histology as assessed on biopsy: Bloom-Richardson grade 1 or 2 Non-lobular invasive histological type carcinoma Oestrogen receptor positive HER2 receptor negative No lymphovascular invasion	Collagen synthesis disease (eg,osteogenesis imperfecta, Ehlers-Danlos syndrome, systemic sclerosis)
	Delayed wound healing after primary treatment
No extensive DCIS (outside tumour size of 2 cm) on mammography or tumour biopsy, including non-mass enhancement on MRI	Previous ipsilateral mastectomy
Clinical node negative on 18-F FDG PET-CT, ultrasound and MRI	Invasive lobular carcinoma or DCIS without invasive cancer
No distant metastasis	MRI absolute contraindications and intolerance
No or mild late toxicity (no grade 2 or higher) from previous breast-conserving therapy	Indication for treatment with neoadjuvant chemotherapy
Adequate understanding of the Dutch language	Legal incapacity
	Poor cosmetic outcome after initial breast-conserving therapy

DCIS, ductal carcinoma in situ; FDG PET-CT, fluorodeoxyglucose positron emission tomography computed tomography; HER2, human epidermal growth factor receptor 2.

### Study outcomes

**Figure 1:**
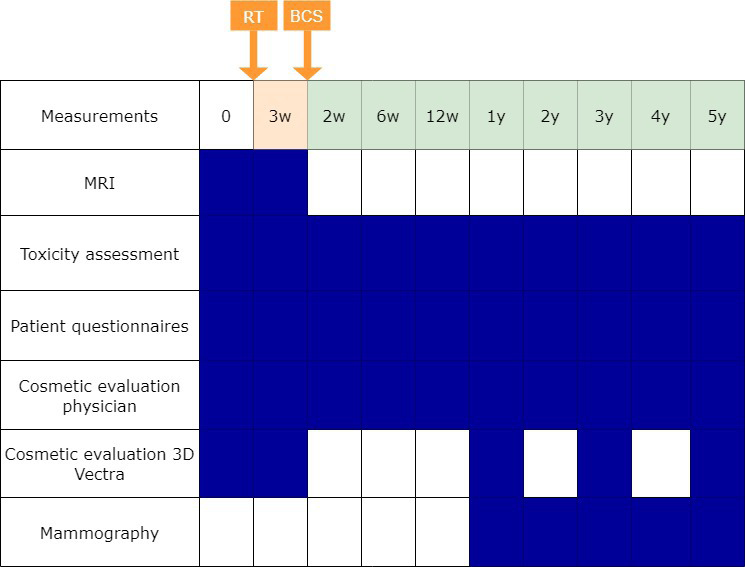
Overview of outcome assessments. Time point in orange after RT. Time points in green after breast-conserving surgery (BCS). Patient questionnaires include evaluation of cosmetic outcomes and QoL. QoL, quality of life; RT, radiotherapy; w, weeks; y, years.

The primary endpoint is the rate of acute ≥grade 2 treatment-induced toxicity within 3 months following preoperative RT, using the Common Terminology Criteria for Adverse Events (CTCAE) V.0 (skin toxicity, breast oedema, breast pain and chest wall pain) and the Clavien-Dindo classification (wound infection). Secondary endpoints are any acute (including grade 1) and late treatment-induced toxicity according to CTCAE V.5.0 and the Clavien-Dindo classification. Radiological response will be assessed using a breast MRI, 3 weeks after RT (preoperatively). Pathological response will be evaluated in the resection specimen. In addition, the mastectomy rate will be assessed 5 years after BCS. PROMs will be evaluated using the European Organization for Research and Treatment of Cancer core-30 and breast cancer-specific questionnaires. Cosmetic outcome will be assessed by the patients using the BREAST-Q questionnaire. The physician will score cosmetic outcome as excellent, good, fair or poor based on changes in breast appearance. Additionally, cosmetic outcome will be evaluated objectively using digital photographs analysed by the BCCT.core software and 3D Vectra XT software. Oncological outcomes will be assessed by evaluating local, regional and distant event rates, and disease-free, breast cancer-specific survival (BCSS) and OS. Disease-free survival is defined from the date of RT to the date of local, regional or distant recurrence. BCSS and OS will be determined from the date of RT to the date of death caused by breast cancer and any cause, respectively. RT-associated (immune-activation/-suppressor) biomarkers in blood and tumour tissue will be explored. An overview of the time points at which the outcomes are assessed is shown in figure 1.

### Procedures

**Figure 2:**
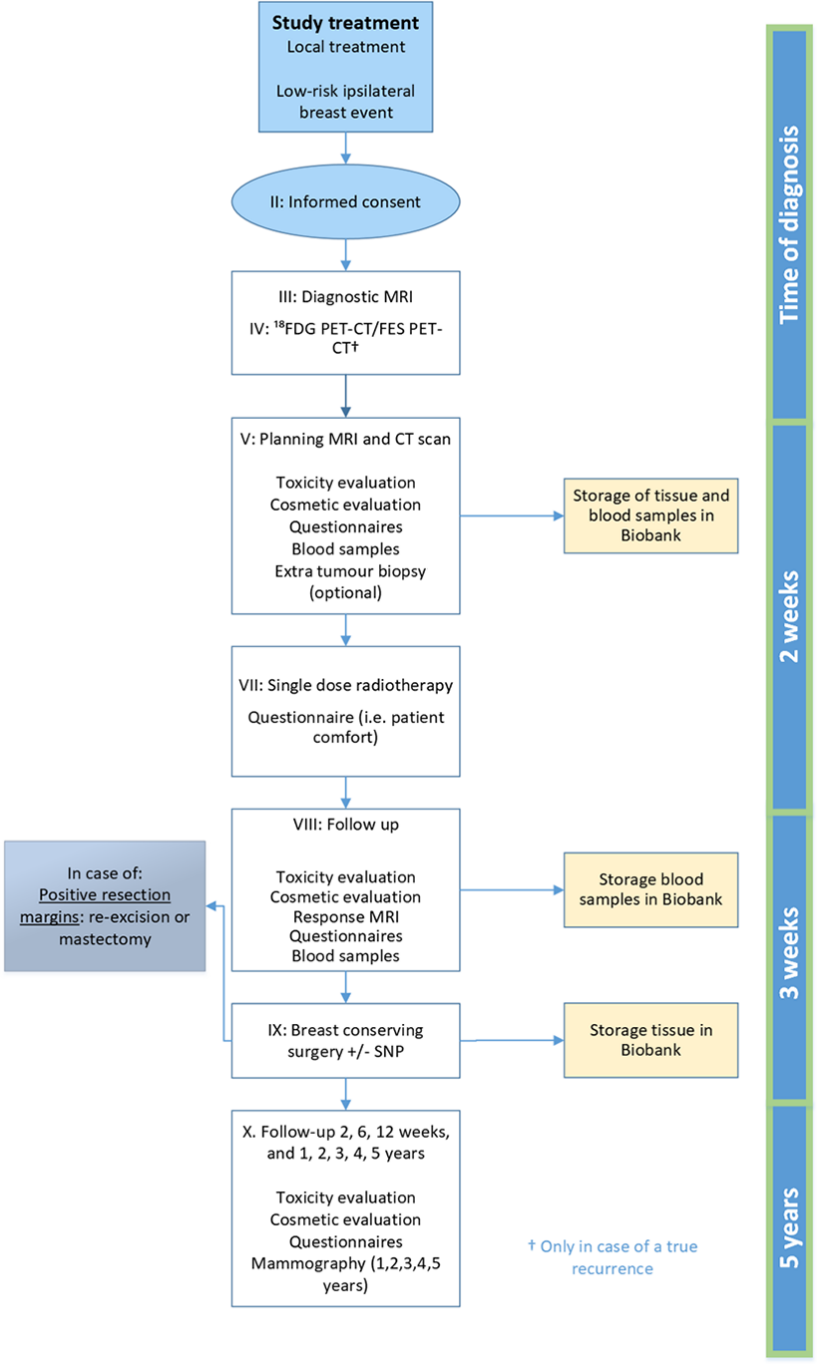
Overview of study procedures. FDG PET-CT fluorodeoxyglucose positron emission tomography computed tomography; FES 18F-Fluoroestradiol; SNP sentinel node procedure

A flow diagram of the study procedures in the REPEAT trial is shown in figure 2. The surgeon, oncologist or nurse practitioner at the outpatient surgery centre will inform patients about the study during their consultation. If an eligible patient expresses interest, the professional will provide written study information and informed consent forms. Subsequently, the patient will have a consultation with a radiation oncologist at the department of radiation oncology for evaluating the inclusion and exclusion criteria. The patient signs the informed consent forms at the department of radiation oncology after a minimum of 2 days to consider study participation.

#### Diagnostic work-up

After informed consent is obtained, an 18F-FDG PET-CT scan will be conducted (if not yet performed) to exclude macroscopic axillary lymph node involvement and distant metastases. If no marker has been inserted in the tumour during the diagnostic biopsy, an MRI compatible clip will be placed in the tumour to allow tumour localisation after neoadjuvant treatment. Additional tumour biopsies will be performed in the same session if separate consent has been obtained.

#### Treatment planning

An MRI with intravenous contrast (ie, gadolinium) and CT will be performed for RT treatment planning in supine position. The gross tumour volume (GTV), which is the breast tumour, is delineated on the simulation MRI. To account for microscopic disease, the GTV is uniformly expanded by 2 cm to create the clinical target volume (CTV), while excluding the first 5 mm below the skin and the entire chest wall including the pectoral muscles, not extending outside of the breast tissue. To account for delineation and setup uncertainties, the planning target volumes (PTV) are generated. The GTV is expanded by 3 mm, and the CTV is expanded by 3 mm (MR-linac) or 5 mm (CBCT-linac) to obtain the PTVs, PTV_GTV_ and PTV_CTV_, respectively.

Treatment techniques consist of intensity modulated RT (IMRT) on an MR-linac. On a conventional linac, IMRT or volumetric modulated arc therapy will be used. A single fraction involves two concomitant RT dose levels: 15 Gy to the PTV_CTV_ and 20 Gy to the PTV_GTV_. Adequate coverage of the target areas is determined by ensuring a mean dose (D_mean_) of 99–101% for PTV_GTV_ and PTV_CTV_, with at least 95% of the prescribed dose delivered to cover 98% of the PTVs. Additionally, restricting the dose to 107% or less in only 2% of the PTV_GTV_ is essential, while adhering to the prescribed doses for organs at risk (table 2). Failure to meet these predefined dosimetric constraints results in exclusion of the patient from the study.

**Table 2:** Dose constraints organs at risk

Ipsilateral breast	D_mean_<5 GyPTV_CTV<25% of total breast volume
Contralateral breast	No constraint, dose as low as possible.
Heart	V _2.8Gy_<10%V _4.7Gy_<5%D_mean_≤1.2 Gy
Lungs	V_16Gy_ < 15 ccD_mean_≤2.66 Gy
Ipsilateral lung	V _6.2Gy_<10%V _12.4Gy_<0.5 cc
Chest wall	D_20cc_ < 16 GyAim for V_20Gy_ < 1 cc*Absolute constraint: V _22Gy_<1 cc, D_max_<30 Gy (cumulative dose)
skin	Dose as low as possible; aim for D_1cc_ < 12 Gy if this is not feasible aim for D_1cc_ < 16 Gy

*according to AAPM criteria, is expected to be feasible in patients with no late toxicity grade 2 or higher

CTV, clinical target volume; D, dose; GTV, gross tumour volume; PTV, planning target volume; V, volume.

#### RT treatment delivery

The single-dose PBI will be delivered within 14 days subsequent to the planning MRI and CT images. MRI-guided RT on an MR-linac will involve repeated breath-hold periods for patients exhibiting substantial tumour movement. Free breathing will be used when tumour position remains unaffected by breathing patterns. Real-time MRI tumour tracking will be used on a MRIdian. On the conventional linac, breath-hold periods will only be used for patients with left-sided tumours for optimal cardiac sparing. On conventional linacs, real-time position management and/or surface guidance will facilitate patient tracking. Additionally, patients will be asked to complete a questionnaire once (5 min) regarding comfort levels during treatment delivery (online supplemental material 1).

#### Preoperative follow-up

Approximately 3 (±one) weeks after single-dose PBI, a diagnostic MRI will be performed just prior to BCS, to assess the acute tumour response after RT. Radiological response is evaluated based on decrease in contrast uptake and decrease in diffusion restriction. The Response Evaluation Criteria in Solid Tumors (RECIST) criteria (V.1.1) are used to monitor tumour response. The MRI will be combined with collection of blood sample for isolation of peripheral blood mononuclear cells.

#### Breast-conserving surgery

BCS will be performed approximately 3 (±one) weeks after single-dose PBI. Axillary staging and treatment will be performed based on the decision of the multidisciplinary tumour board of the respective hospital according to standard of care. All patients will receive a prophylactic single dose (2 g) of Cefazolin 30 min prior to surgery, to prevent any wound infection. The surgical specimen will be examined by an experienced breast pathologist. Viable tumour cells will be assessed using H&E staining alongside the evaluation of cytokeratin antibody activity. Pathological response is assessed according to the residual cancer burden (RCB). RCB is calculated as a continuous variable (RCB calculator https://www3.mdanderson.org/app/medcalc/index.cfm?pagename=jsconvert3) and classified into four RCB classes indicating progressively larger residual disease burden^16^:

- RCB-0 (RCB score 0, equivalent to pathologic complete response).
- RCB-I (RCB score ≥0–1.36, minimal burden).
- RCB-II (RCB score 1.37–3.28, moderate burden).
- RCB-III (RCB score >3.28, extensive burden).

The following parameters are required from pathologic examination to calculate RCB: the largest two dimensions (mm) of the residual tumour in the breast, average percentage overall tumour cellularity (invasive and in situ), average percentage of the carcinoma in and outside the tumour bed that is in situ, number of positive lymph nodes and size of the largest axillary metastasis (mm).

Furthermore, the frequency of patients presenting with DCIS within the surgical specimen will be documented. India ink will be used to mark the surface of the excised specimen to evaluate the surgical margins. Surgical margins will be described as the minimal microscopic tumour-free margin of invasive and in situ carcinoma (in mm) in the direction of the nearest surgical margin. In the case of involved surgical margins, a re-excision will be performed.

#### Follow-up

Patients with an indication for adjuvant endocrine therapy will start this treatment following BCS on prescription of the surgeon or medical oncologist. If microscopic findings (eg, Bloom-Richardson grade 3, tumour size >2 cm, triple negative or HER2-positive breast cancer) in the surgical specimen necessitate additional adjuvant systemic therapy, patients will receive appropriate treatment according to Dutch national guidelines.^17^ Annual clinical consultations, including a physical examination, with the attending radiation oncologist will be conducted up to 5 years following the second BCS. During these consultations, the radiation oncologist or physician assistant will assess both toxicity and cosmetic outcomes (figure 1). Additionally, patients will complete questionnaires (20 min) related to QoL and cosmetic outcomes at the following time points: baseline, preoperatively, 2 weeks, 6 weeks, 12 weeks and annually until 5 years after RT. Objective evaluation of cosmetic outcomes will be conducted through the capture of digital photographs at baseline, preoperatively, 1 year, 3 years and 5 years post RT. Consistent with the standard of care, radiological follow-up will comprise annual mammograms during the initial 5-year period similar to standard follow-up.

### Biobank

The diagnostic biopsy, the surgical specimen and blood samples will be stored (after additional informed consent by the patient) for a duration of 25 years at the Amsterdam UMC Central Biobank. Optional informed consent will be requested from the patient for two or three 14G tumour biopsies in order to investigate gene expression profiling in breast tumour tissue.

### Ethics and dissemination

This study will be conducted according to the Declaration of Helsinki (Fortaleza, Brazil, October 2013) and the Dutch Medical Research Involving Human Subjects Act (http://www.ccmo.nl). The study protocol has received approval from the Medical Research Ethics Committee of the Amsterdam University Medical Centre (NL85983.018.24), and has been registered in an international trial registry (ClinicalTrial.gov: NCT06640881). Approval has also been granted by the institutional review board of each participating centre. Subsequent to a written and oral explanation of the study, all participants are required to provide written informed consent prior to inclusion (online supplemental material 2). The results will be submitted/published in (peer-reviewed) journals and presented at scientific meetings.

### Patient and public involvement

Two patients advocates from the Dutch Breast Cancer Association reviewed the first draft of the study protocol. They were satisfied with the selected outcome measures and highlighted the importance of collecting oncological outcomes and QoL data. Additionally, they supported the proposal to make the additional tumour biopsy optional, aiming to minimise the treatment burden for participants. Newsletters including the results will be provided to participating patients.

### Quality assurance

In order to ensure the quality and integrity of the research data, an independent and qualified monitor will conduct centralised study monitoring at the Clinical Monitoring Centre of the Amsterdam UMC. The monitoring activities will adhere to national guidelines governing quality control for Dutch University Medical Centres.^18^

### Statistical analysis

The sample size calculation is based on the primary endpoint of the rate of grade 2 or higher acute treatment-associated toxicity within 90 days (skin toxicity, breast oedema, breast pain, chest wall pain, wound infection). We expect that 30% of our patients will experience ≥grade 2 acute toxicity following preoperative RT. An unacceptable proportion is considered if acute toxicity is observed in 50% or more of the patients. If, during the study duration, more than 50% of the first 10 patients develop ≥grade 2 acute toxicity, the study will be terminated. Therefore, based on the sample size calculation, which ensures that the CI does not encompass an incidence of 50%, a statistical power of 80% and a two-sided alpha of 0.05, a sample size of 21 patients is determined. Adjusting for a 15% allowance to accommodate patients deemed ineligible, the total required sample size is 25.

The assessment of the proportion of patients experiencing ≥grade 2 acute toxicity will be evaluated, and a 95% CI will be calculated. Grade 1 acute and late treatment-induced toxicity, PROMs and cosmetic results will be compared with baseline using linear mixed models for repeated measures. Radiological and pathological response, mastectomy rate, patient comfort during RT treatment delivery and oncological outcomes will be presented using descriptive statistics. The Kaplan-Meier method will be employed to quantify disease-free, breast cancer-specific and OS. Baseline T-cell levels will be analysed across patients to assess interpatient variability and intrapatient changes between baseline and post-treatment timepoints. Overlap in variance among different response markers will be explored. Additionally, the reproducibility of potential response markers, if applicable, will be investigated.

## Discussion

The REPEAT trial aims to investigate the feasibility of second BCT with preoperative PBI among patients with a low-risk IRBE. The feasibility will be assessed based on the incidence of grade 2 or higher acute toxicity induced by RT and surgery. Additionally, the trial will assess PROMs, cosmetic outcomes, oncological endpoints and RT-associated (immuno) biomarkers in both blood and tumour tissue.

Numerous randomised controlled trials (RCTs) have compared BCT and mastectomy, demonstrating similar oncological outcomes in primary breast cancer.^19^ In addition to the excellent survival and recurrence rates, BCT offers an advantage in terms of QoL.^20^,^22^ Patient undergoing BCT have reported improved body image, future perspectives, psychosocial and sexual well-being, with these improvements showing a gradual increase over time.^20^,^22^ It is therefore obvious to consider a second BCT instead of a standard salvage mastectomy in the event of a local recurrence. Advancements in RT techniques over the last decades, such as CT-guided and MR-guided RT and (ultra-) hypofractionation, allow more precise treatment delivery and a lower total dose, respectively, resulting in reduced toxicity. Consequently, the interest in second BCT is increasing.^23^ A systematic review conducted by Walstra *et al* showed that second BCT is feasible.^9^ The weighted estimates for grade 3 and 4 toxicity were 11% for WBI, 9% for external-beam PBI, 9% for interstitial brachytherapy and 18% for intraoperative radiation therapy (IORT). These results suggest a low incidence of adverse events comparable to postoperative RT.^5 9 24^ The cosmetic results were excellent or good in 29–100% of the patients.^9^ Furthermore, patients reported significantly better QoL scores for body image and overall QoL after second BCT compared with those treated with salvage mastectomy. However, data on toxicity, cosmetic outcome and QoL after second BCT were limited, and all available data on second BCT are from retrospective cohort studies. In contrast to primary breast cancer, no RCTs have been conducted comparing second BCT with mastectomy in patients with IRBE, due to the low incidence rate.

The majority of studies investigating reirradiation after BCS primarily focus on brachytherapy.^9^ Additionally, in previous studies on second BCT, whole breast reirradiation was more commonly investigated compared with PBI. In contrast to the primary setting, no RCTs have been conducted to date comparing re-WBI and re-PBI. Re-PBI is increasingly recognised for its benefits, as the localised treatment reduces radiation exposure to the whole breast and associated side effects.^9^ The recent study of Hannoun-Levi *et al* indicates that re-PBI can effectively manage recurrences with acceptable toxicity profiles, further supporting its feasibility in clinical settings.^6^

The selection criteria for patients eligible for second BCT are critical for optimising outcomes. Previous studies provide valuable guidelines for selecting patients suitable for second BCT, emphasising the importance of the interval since the initial treatment.^6 10 25^ There is no consensus on the optimal timing or interval between the primary tumour and recurrence. A shorter interval to the first recurrence is associated with a higher risk of subsequent local and distant recurrences. The eligibility criteria from Gentilini *et al* are tumour size ≤2 cm, a clinically negative axilla, any age and candidates to receive BCT.^25^ These criteria help in identifying patients who are most likely to benefit from second BCT; however, prospective studies are warranted. In addition, understanding the prognostic differences between true recurrences and second primary tumours is crucial for tailoring treatment. Studies suggest that true recurrences generally have a worse prognosis compared with second primary tumours. The recently developed Groupe Européen de Curiethérapie-European Society for Therapeutic Radiology and Oncology TAM score (score based on the combination of time interval between first and second breast surgery [T] and accelerated PBI [A] and molecular [M] classifications) could aid in the decision-making process for a second BCT.^6^ Moreover, tumour-infiltrating lymphocytes (TILs) and circulating tumour DNA (ctDNA) could provide prognostic insights into outcomes.^26^,^28^ RT can modulate the immune response as measured in TILs.^28^ Elevated ctDNA levels post RT could indicate a higher risk of recurrence, providing a valuable biomarker for prognosis.^26 27^ However, data on the value of TILs and ctDNA in patients treated with RT are scarce and require further investigation.

The presence of the tumour is in situ during PBI, allowing for more precise delineation of target volumes and reduced interobserver variability.^13 29^ Both factors result in smaller irradiated volumes, which allow a higher prescribed dose to be delivered on the tumour in a reduced number of fractions, potentially lowering radiation-associated toxicity and improving cosmetic results.

A recent systematic review of studies on preoperative PBI in primary breast cancer showed that acute toxicity resulting from preoperative RT primarily manifests as grade 1 (19–34%), grade 2 (0–10%) skin toxicity; no grade 3 or higher was reported. The most common late toxicities are fibrosis grade 1 (56–100%) and 2 (9%), breast discomfort/pain grade 1 (13–58%) and 2 (6–13%) and breast oedema grade 1 (31%).^12^ Cosmetic outcomes were assessed as good or excellent by 78–100% of the patients. Concerns have been raised regarding the increased risk of surgical complications after preoperative RT. However, recent studies suggest that a short interval between RT and BCS does not necessarily increase postoperative complications.^12^ In the SIGNAL trial, only 1/52 patients experienced a wound infection after single-dose preoperative PBI followed by BCS after 1 week.^30^ In addition, single-dose preoperative PBI reduces treatment burden for patients and streamlines healthcare logistics in comparison to multiple (ie, 5–23) postoperative re-PBI fractions. Furthermore, it is more cost-effective and sustainable by minimising visits and potentially decreasing side effects and subsequent treatments, thereby lowering overall healthcare costs. This preoperative approach could, after proven effectiveness and limited toxicity, be extrapolated to second BCT in patients with an IRBE with low-risk characteristics.

The value of a repeat sentinel lymph node biopsy (SLNB) as a staging tool in patients with an IRBE is disputable. The risk of axillary lymph node metastasis in patients with an IRBE after previous BCS and SLNB is 26%.^31^ A meta-analysis showed that a repeat SLNB has a negative predictive value of 96.5% and a false negative rate of 4.1% for tumour negative nodes.^32^ In the SNARB study, 515 patients with an IRBE underwent repeat SLNB for axillary staging after a median recurrence interval of 10.5 years (0.4–31.8).^33^ In the primary setting, 203 (39.4%) patients underwent an SLNB and 278 (54%) underwent axillary lymph node dissection. The repeat SLNB could not identify the sentinel node in 239 (46.4%) patients.^33^ No significant difference was found in the 5-year recurrence-free survival between patients with an unsuccessful, tumour-positive or tumour-negative SLNB (p=0.543).^33^ Additionally, a recent retrospective cohort of 119 patients with an IRBE with negative lymph nodes on 18F-FDG-PET/CT who underwent repeat SLNB showed that repeat SLNB was successful in 66% of the patients and 8% had a tumour-positive sentinel node.^34^ The 10-year OS was comparable across patients with tumour-positive, tumour-negative and unsuccessful repeat SLNB (89% vs 89% vs 95%, p=0.701). Consequently, in the REPEAT study, a multidisciplinary tumour board will determine the axillary staging method. This also simplifies the treatment process with preoperative PBI and could reduce SLNB-associated morbidities.

Possible limitations of this prospective multicentre study are the relatively short follow-up. The reason for the 5-year follow-up is because of the primary outcome acute toxicity and the feasibility design of this study. Second, the interval between RT and surgery is too short to achieve a pathological complete response. Results from the ABLATIVE trial showed that the rate of PCR is higher after 8 months (48%) compared with 6 months (33%).^14^ This suggests, although patient numbers were small, that the RT may still kill tumour cells after 6 months. To ensure oncological safety in these patients with recurrent breast cancer, we chose an interval of 3 weeks for this feasibility study.

## Supplementary material

10.1136/bmjopen-2024-096510 online supplemental file 1

10.1136/bmjopen-2024-096510 online supplemental file 2
