# Supplementary material for: Preoperative partial breast reirradiation and repeat breast-conserving surgery in patients with recurrent breast cancer: the prospective single-arm REPEAT trial – a study protocol
Source: BMJ Open. 2025 Jul 18;15(7):e096510. doi: 10.1136/bmjopen-2024-096510 (PMC12273098; doi:10.1136/bmjopen-2024-096510)
Supplement: online supplemental file 1 [file bmjopen-15-7-s001.docx]

**Date of Completion:** __ / __ / __
**Study:** ..........................................................
**Radiation Indication MRIdian:** .....................
**Participant Code:** ..............................................

Due to the intensive treatment on this new device, we would like to evaluate how you experienced it. Please indicate to what extent the following questions apply to you.

| **During Preparation:** | | **Not at all** | **A little** | **Moderately** | **Very much** |
| --- | --- | --- | --- | --- | --- |
| 1. | Was the explanation about the preparation procedure and treatment understandable? | 1 | 2 | 3 | 4 |
| 2. | Were you well-prepared by the radiation specialist for the expected side effects? | 1 | 2 | 3 | 4 |
| 3. | Did you find the CT scan and MRI scan on the radiation device burdensome? | 1 | 2 | 3 | 4 |
| 4. | Was the waiting time between the first consultation with the radiotherapist and the start of treatment acceptable? | 1 | 2 | 3 | 4 |
| **During Treatment:** | | | | | |
| 5. | Did you feel anxious while lying in the tunnel? | 1 | 2 | 3 | 4 |
| 6. | Was the time you spent on the radiation device acceptable? | 1 | 2 | 3 | 4 |
| 7. | Were the instructions you received (via headphones and/or screen) understandable? | 1 | 2 | 3 | 4 |
| 8. | Did you find the staff's guidance on the radiation device professional? | 1 | 2 | 3 | 4 |
| 9. | Did the symptoms caused by your illness increase during the radiation? | 1 | 2 | 3 | 4 |

Please turn to the next page.

| **During treatment:** | | **Not at all** | **A little** | **Moderately** | **Very much** |
| --- | --- | --- | --- | --- | --- |
| 10. | Did you experience the following while lying on the MRI? |  |  |  |  |
|  | - Feeling of warmth | 1 | 2 | 3 | 4 |
|  | - Cold | 1 | 2 | 3 | 4 |
|  | - Dizziness | 1 | 2 | 3 | 4 |
|  | - Tingling | 1 | 2 | 3 | 4 |
|  | - Metallic taste | 1 | 2 | 3 | 4 |
|  | - Light flashes | 1 | 2 | 3 | 4 |
|  | - Noise | 1 | 2 | 3 | 4 |
| **The following questions relate to your active role during the breath-guided radiation:** | | | | | |
| 11. | Did you find it difficult to position the target area by holding your breath? | 1 | 2 | 3 | 4 |
| 12. | Did you find it burdensome to see your tumor during the radiation? | 1 | 2 | 3 | 4 |
| 13. | Did you appreciate having an active role during the radiation? | 1 | 2 | 3 | 4 |
| 14. | Were you concerned about your role during the radiation in the past period? | 1 | 2 | 3 | 4 |

Thank you very much for your cooperation.
