# Supplementary material for: Preoperative partial breast reirradiation and repeat breast-conserving surgery in patients with recurrent breast cancer: the prospective single-arm REPEAT trial – a study protocol
Source: BMJ Open. 2025 Jul 18;15(7):e096510. doi: 10.1136/bmjopen-2024-096510 (PMC12273098; doi:10.1136/bmjopen-2024-096510)
Supplement: online supplemental file 2 [file bmjopen-15-7-s002.docx]

**Participant Information for participation** **in medical-scientific research**

**Single re-irradiation for breast cancer prior to a second breast-conserving surgery (REPEAT trial)**

*Official title (in Dutch): Eenmalige preoperatieve partiële borstbestraling en tweede borstsparende operatie bij patiënten met een ipsilateraal borstkankerrecidief. (Single preoperative partial breast irradiation and second breast-conserving surgery in patients with ipsilateral breast cancer recurrence)*

**Introduction**

Dear Madam,

With this information letter, we would like to ask if you want to participate in medical-scientific research. Participation is voluntary. You are receiving this letter because breast cancer has been found in you for which you can undergo breast-conserving treatment again.
You can read here what kind of research it is, what it means for you, and what the advantages and disadvantages are. This is a lot of information. Would you like to read the information and decide if you want to participate? If you want to participate, you can fill out the form found in Appendix D.

**Ask your questions**

You can make your decision with the information you find in this information letter. In addition, we recommend that you do this:

- Ask questions to the researcher who gives you this information.

- Talk with your partner, family, or friends about this research.

- Ask questions to the independent expert. For contact details, see Appendix A

- Read the information at [www.rijksoverheid.nl/mensenonderzoek](http://www.rijksoverheid.nl/mensenonderzoek).

1. **General information**

Amsterdam UMC has set up this research. Hereafter, we will always refer to Amsterdam UMC as the 'sponsor'. Researchers, who may also be doctors and research nurses, carry out the research in various hospitals.

Participants in medical-scientific research are often called subjects. Both patients and healthy people can be subjects. About 25 subjects are expected to participate in the Netherlands. The medical ethics review committee of Amsterdam UMC has approved this research.

1. **What is the purpose of the research?**

The purpose of the research is to find out how safe a single re-irradiation of part of a breast is, prior to a second breast-conserving surgery. We are investigating the side effects of a second breast-conserving surgery when breast cancer returns.

1. **What is the background of the research?**

Patients with breast cancer usually receive breast-conserving treatment. This means that a breast-conserving surgery takes place within 4 weeks after diagnosis. In some patients, one or more lymph nodes are also removed from the armpit to rule out tumor presence. This is called a 'sentinel node procedure'. On average, radiation therapy of the entire or part of the breast begins 5 weeks after surgery. In total, radiation is given between 5 to 20 times over a period of 1 to 5 weeks.

When breast cancer returns, the standard treatment is removal of the entire breast. A second breast-conserving surgery with re-irradiation is increasingly being done. In this study, we give the radiation not after, but before the breast-conserving surgery. And we irradiate just once. This is done with a high dose. We irradiate very precisely because the tumor is still present. The surgery takes place three weeks after the radiation. This is different from the standard treatment. We expect fewer side effects from the single, partial breast irradiation. This is because we only irradiate once in a targeted manner, compared to 5 to 20 partial or whole breast irradiations in standard treatment. It also prevents the entire breast from being removed.

In earlier research, we have seen that a second breast-conserving therapy is safe in women with a low risk of disease recurrence. The side effects have been mild so far. Also, radiation before surgery in previous research in women with breast cancer shows that this gives a good cosmetic result in most women.

1. **How does the research proceed?**

If you choose to participate in this study, we will first check if you meet certain criteria. These criteria have been established to ensure the safety of patients participating in this study.

For the research, you need to come to [HOSPITAL NAME] several times: before the preparation of radiation, for the radiation, between the radiation and the breast-conserving surgery, and after the breast-conserving surgery.

**Prior to radiation**

A standard CT scan, which is always done before radiation, and again an MRI scan (now in radiation position) will be performed.

Subsequently, six tubes of blood will be taken to conduct research on certain substances in the blood that can provide information about specific characteristics of the tumor and sensitivity to radiation, so-called marker substances. In total, we will take 52 mL of blood from you. This amount does not cause problems. These blood tests may possibly improve breast cancer treatments in the future. Each blood draw will be combined with another appointment as much as possible.

In women with a small tumor, a small clip is placed in the tumor during standard treatment, a so-called marker. If this has not been inserted in participants of this study, then this marker will be inserted prior to the partial breast radiation. This marker is inserted under local anesthesia by a radiologist. This is necessary to accurately determine the area of the tumor during breast-conserving surgery. This marker will also be removed with the breast-conserving surgery. The placement of the marker will be combined with taking 2 or 3 pieces of tissue from the breast tumor (depending on which hospital you are being treated in). These tissue pieces will be used to study the effect of radiation. If a marker has already been inserted, a separate appointment will be made for taking some pieces of breast tumor tissue under local anesthesia. You can give permission for this on the consent form. Don't you give permission? Then you can still participate in this research. You will receive the same care.

**The radiation**

The partial breast radiation will be given once. In standard treatment, radiation is given multiple times. As a result, the total treatment with radiation in this study is shorter than in standard treatment.

**After radiation**

After the radiation, an MRI scan will be made after 3 weeks to check how the tumor responds to the radiation. This MRI scan is combined with an appointment at the Radiotherapy outpatient clinic. Six tubes of blood will also be taken during this MRI scan.

**The surgery**

The breast-conserving surgery (and possible sentinel node procedure) will take place 3 weeks after the partial breast radiation. If the treatment team decides that the sentinel node is necessary for you, this will take place simultaneously with the breast-conserving surgery. The portion removed by the surgeon will, if you give permission for this, be stored in the Central Biobank of Amsterdam UMC, location VUmc. This will be used for future research on breast cancer and radiation.

The surgery does not require extra time investment from you compared to the surgery in standard treatment.

Just as with standard treatment, some patients are eligible for hormonal therapy. Those concerned will receive this therapy immediately after surgery. Your treating physician will discuss this with you; this does not change due to participation in the study.

**After breast-conserving surgery**

After the surgery, you will remain under follow-up at the Radiotherapy outpatient clinic for 5 years, and you will also come for check-ups with the surgeon several times. During these check-ups, a mammogram will be made annually, and a physical examination will be performed.

***What is different from regular care?***

- 1 radiation session (takes on average 45 minutes) instead of 5, 15, or 20 times (takes 20 to 30 minutes each time);
- 1 extra MRI scan with blood draw (takes 30 to 45 minutes per MRI and blood draw);
- If necessary, placement of a marker in the breast tumor and taking tissue samples (takes about 30 minutes);
- Two times taking six tubes (52 mL) of blood (takes about 10 minutes per draw);
- Filling out a questionnaire about the radiation (takes 5 minutes);
- For 5 years, filling out questionnaires about quality of life, side effects, and cosmetic result (10 times, takes about 20 minutes per questionnaire) and taking digital photos for cosmetic result (5 times, takes about 15 minutes each time).

An overview of hospital visits and questionnaire moments during participation in this study can be found in Appendix C.

1. **What agreements do we make with you?**

We want the research to go well. Therefore, we make the following agreements with you:

- You report participation in other medical-scientific research.
- You come to every appointment.
- You contact the researcher in these situations:
  - You want to start using other medications. This also applies to homeopathic remedies, natural medicines, vitamins, or over-the-counter drugs.
  - You are admitted to a hospital or receive treatment.
  - You suddenly have health problems.
  - You no longer want to participate in the research.
  - Your phone number, address, or email address changes.

1. **What side effects, adverse effects, or discomforts might you experience?**

**Treatment**

The single radiation can cause side effects. Just as with standard radiation for breast cancer, the following side effects can occur in two phases. In the early phase, up to about 6 weeks after radiation, skin complaints can occur in particular. This can manifest as redness, itching, and swelling of the breast. The complaints are treated with an ointment and disappear on their own within 6 weeks. In the late phase, from about 3 months after radiation, scar tissue can form in the breast. There is a small chance of lung irritation. In the long term, there is a small chance of developing heart conditions. This single radiation is given very locally to the tumor, so we expect the chance of these side effects to be lower than with standard treatment. The severity of these complaints and the chance of developing them can vary per patient. Surgery after radiation leads to the same possible risks as with standard treatment. These are chances of post-operative bleeding, wound infection, or bruising.

1. **What are the advantages and disadvantages if you participate in the research?**

Participating in the research can have advantages and disadvantages. Below we list them. Think about this carefully, and talk about it with others. The single partial breast radiation can ensure that in this study, you can undergo a breast-conserving operation, whereas according to standard treatment, you would be eligible for removal of the entire breast. If according to standard treatment, you could undergo a second breast-conserving operation, the advantage of the research is that you will be irradiated only once instead of 5 to 20 times in standard treatment. Based on the results of the earlier research on single partial breast radiation followed by a second breast-conserving operation, we expect that this research treatment works just as effectively against breast cancer as the standard treatment. Furthermore, we expect that you will have fewer side effects from the single radiation. But that is not certain. Your side effects can also remain the same or return or worsen at any time during this research.

Participating in the research can have these disadvantages or consequences:

- You may experience side effects or adverse effects of this radiation treatment, as described in paragraph 6.
- Participating in the research costs you extra time.
- You must comply with the agreements associated with the research.
- It is possible that something is accidentally discovered on an MRI scan that is not directly relevant to the research but is relevant to your health or that of your family members. See also paragraph 10 about unexpected discoveries.

Just as with standard treatment, there is a risk that breast cancer will return again in your body. In case of a possible local recurrence of breast cancer in the same breast, you cannot, just as after standard treatment, be irradiated again in the same breast.

*Don’t want to participate?*

You decide yourself whether to participate in the research. Don’t want to participate? Then you will receive the standard treatment for breast cancer. Your doctor can tell you more about the treatment options available. And about their advantages and disadvantages.

1. **When does the research stop?**

The researcher will let you know if there is new information about the research that is important for you. The researcher will then ask you if you will continue to participate.

In these situations, the research stops for you:

- All investigations according to the schedule are completed.
- You want to stop participating in the research yourself. You may do this at any moment. Inform the researcher immediately. You do not have to tell why you are stopping. You will then receive the usual treatment for breast cancer again.
- The researcher finds it better for you to stop. The researcher will still invite you for a follow-up check.
- One of the following institutions decides that the research must stop:
  - Amsterdam UMC
  - the government, or
  - the medical ethics committee that evaluates the research.

*What happens if you stop participating in the research?*

The researchers use the data and body material (blood and tumor tissue) that have been collected up to the moment of stopping. If you wish, collected body material can be destroyed. Let the researcher know this.

The entire research is completed when all participants are finished.

1. **What happens after the research?**

After your participation and the processing of all data, the researcher will let you know what the main outcomes of the research are. Do you not want to know this? Then tell the researcher. He/she will then not tell you.

1. **What do we do with your data and body material?**

If you participate in the research, you also give permission to collect, use, and store your data and body material.

*Which data do we store?*

We store these data:

- your name

- your gender

- your address

- your date of birth

- data about your health

- (medical) data that we collect during the research

*Which body material do we store?*

We collect, use, and store tubes of blood and tumor tissue.

*Why do we collect, use, and store your data and body material?*

We collect, use, and store your data and your body material to be able to answer the questions of this research. And to be able to publish the results.

*How do we protect your privacy?*

To protect your privacy, we give your data, images, and your body material a code. We put only this code on all your data and body material. The key to the code is kept in a secure place in the hospital. When we process your data and body material, we always use only that code. Also, in reports and publications about the research, no one can trace that it was about you.

*Who can see your data?*

Some people can see your name and other personal data without a code. These can be data specifically collected for this research, but also data from your medical record.

These are people who check whether the researchers are conducting the research properly and reliably. These people can access your data:

- Members of the committee that monitors the safety of the research.
- An inspector who works for the sponsor.
- National and international regulatory authorities. For example, the Healthcare and Youth Inspectorate.

These people keep your data confidential. We ask for your permission for access by these people. The Healthcare and Youth Inspectorate can access your data without your permission.

*How long do we keep your data and body material?*

We keep your data for 25 years at Amsterdam UMC. We also store your body material at Amsterdam UMC. It is kept for a maximum of 25 years to be able to perform new determinations on it during this research that are related to this research. As soon as this is no longer necessary, we destroy your body material.

*May we use your data and body material for other research?*

Your collected data and your (remaining) body material may also be important for other scientific research by the research team in the field of breast cancer and/or the further development of radiation. For this purpose, your data and body material will be stored for 25 years at Amsterdam UMC. In the consent form, you indicate whether you agree to this. Don't you give permission? Then you can still participate in this research. You will receive the same care.

*What happens with unexpected discoveries?*

During the research, we may accidentally find something that is not directly relevant to the research but is relevant to your health. The researcher will then contact your general practitioner. You will then discuss with your general practitioner or specialist what needs to be done. The costs of this fall under your own health insurance. You give permission for informing your general practitioner or specialist with the form.

*Can you withdraw your consent for the use of your data again?*

You can withdraw your consent for the use of your data at any time. Tell this to the researcher. This applies to the use in this research and for use in other research. But note: if you withdraw your consent, and researchers have already collected data for research, they may still use this data. For your body material, the researchers will destroy it after you withdraw your consent. But have measurements already been done with your body material? Then the researcher may continue to use the results.

*Do you want to know more about your privacy?*

- Do you want to know more about your rights when processing personal data? Look at.
- Do you have questions about your rights? Or do you have a complaint about the processing of your personal data? Please contact the person responsible for processing your personal data. For your research, that is:
  - Amsterdam UMC. See Appendix A for contact details and website.
- If you have complaints about the processing of your personal data, we recommend that you first discuss these with the research team. You can also go to the Data Protection Officer of [INSTITUTION NAME]. Or you can file a complaint with the Dutch Data Protection Authority.

*Where can you find more information about the research?*

On the following website(s), you can find more information about the research: <https://www.toetsingonline.nl>. You can find the research by searching for NL85983.018.24.

1. **Do you receive compensation if you participate in the research?**

The extra tests and treatment for the research cost you nothing. You also do not receive compensation if you participate in this research. However, you will receive compensation for your (extra) travel and parking costs if these are not reimbursed by your health insurer.

1. **Are you insured during the research?**

Insurance has been taken out for everyone who participates in this research. The insurance pays for damage caused by the research. But not for all damage. In **Appendix B**, you will find more information about the insurance and the exceptions. It also states to whom you can report damage.

1. **We inform your general practitioner and your treating specialist**

The researcher sends your general practitioner and your treating specialist a letter to let them know that you are participating in the research. This is for your own safety.

1. **Do you have questions?**

You can ask questions about the research to the researcher. Do you want advice from someone who has no interest? Go to Prof. Dr. C. van Zuijlen. She knows a lot about the research but does not work on this research.

Do you have a complaint? Discuss this with the researcher or the doctor treating you. Would you rather not? Go to the complaints committee of your hospital. In Appendix A, you can find where to find them.

1. **How do you give consent for the research?**

You can first think calmly about this research. You have a reflection period of at least 2 days. Afterward, you tell the researcher whether you understand the information and whether or not you want to participate. Do you want to participate? Then fill in the consent form that you find with this information letter. You and the researcher will both receive a signed version of this declaration of consent.

**Thank you for your time.**

1. **Appendices to this information**

A. Contact details

B. Information about the insurance

C. Research procedures schedule

D. Consent form(s)

**Appendix A: contact details for [INSTITUTION NAME]**

**Principal Investigator [INSTITUTION NAME]**

Name: xx

Phone: xx

Email: xx

**Principal Investigator Amsterdam UMC**

Name: Dr. H.J.G.D. (Desirée) van den Bongard, radiation oncologist

Phone: Via 020-4441571

Email: [h.j.vandenbongard@amsterdamumc.nl](mailto:h.j.vandenbongard@amsterdamumc.nl)

**Coordinating Investigator Amsterdam UMC**

Name: Drs. L.F. (Lisca) Wurfbain, physician-researcher

Phone: Via 06-25414750

Email: [l.f.wurfbain@amsterdamumc.nl](mailto:l.f.wurfbain@amsterdamumc.nl)

**Independent expert**

Name: Prof. dr. C. (Lia) van Zuijlen, medical oncologist

Phone: Via 020-4445152

Email:

**Complaints**

If you have complaints, you can contact [details of complaint mediation participating hospital]

Data Protection Officer of [INSTITUTION NAME]

If you are dissatisfied with how your privacy is handled, you can file a complaint with the Data Protection Officer [contact details Data Protection Officer participating center] or the Data Protection Officer of Amsterdam UMC, location VUmc ([privacy@amsterdamumc.nl](mailto:privacy@amsterdamumc.nl)). You can also contact the Dutch Data Protection Authority.

For more information about your rights: The organization Amsterdam UMC is the data controller. Website: [www.amsterdamumc.nl](http://www.amsterdamumc.nl) Phone number: 020-5669111/ 020-444 4444.

**Appendix B: information about the insurance**

The sponsor Amsterdam UMC has taken out insurance for everyone who participates in the research. The insurance pays for the damage you have because you participated in the research. This concerns damage you get during the research, or within 4 years after the end of your participation in the research. You must report damage within these 4 years to the insurer.

Do you have damage from the research? Report this to this insurer:

The insurer of the research is:

Insurer name: Centramed B.A.

Address: PO Box 7374

2701 AJ Zoetermeer

Phone number: 070 301 70 70

Email: [info@centramed.nl](mailto:info@centramed.nl)

Policy number: 624.100.044

The insurance provides coverage of € 650,000 per subject with a maximum of € 5,000,000 for the entire research and € 7,500,000 for damage resulting from medical-scientific research that is reported per insurance year.

Please note: the insurance does **not** cover the following damage:

- Damage due to a risk about which we informed you in this letter. But this does not apply if the risk turned out to be greater than we thought in advance. Or if the risk was very unlikely.
- Damage to your health that would also have occurred if you had not participated in the research.
- Damage that occurs because you did not follow instructions or directions correctly.
- Damage to the health of your children or grandchildren (unless the research specifically relates to an existing pregnancy or birth, or to embryos with the aim of establishing a pregnancy - in that case, damage to the child/children concerned is covered).
- Damage from an existing treatment method. Or from research into an existing treatment method.

These provisions are in the 'Decree on Compulsory Insurance for Medical-Scientific Research with Human Beings 2015'. This decree is in the government's Law Database.

Furthermore, the subject is requested to contact the Principal Investigator Amsterdam UMC, Dr. H.J.G.D. van den Bongard, radiation oncologist via 020-4441571 in this regard.

**Appendix C: Research procedures schedule**

**Appendix D - consent form subject**

Belonging to

**Single re-irradiation for breast cancer prior to a second breast-conserving surgery (REPEAT study)**

- I give the researcher permission to inform my general practitioner (GP) and treating specialist that I am participating in this study.
- I give the researcher permission to request information from my GP and treating specialist about the current breast cancer diagnosis, medication use, and other relevant health issues.
- I give the researcher permission to provide my GP or specialist with information about unexpected findings from the study that are relevant to my health.
- I give the researchers permission to collect, store, and use my data and/or biological samples. The researchers will only do this to answer the research question of this study. I understand that my data and biological samples will be stored for 25 years.
- I understand that for the purpose of monitoring the study, certain individuals may review all of my data. These individuals are listed in this information letter. I give these individuals permission to review my data for this monitoring purpose.
- Please check "Yes" or "No" in the table below:

| I consent to the storage of my data for use in other research, as described in the information letter. | Yes ☐ | No☐ |
| --- | --- | --- |
| I consent to the collection of additional tissue samples from the breast tumor to analyze the effects of radiation (even if this requires an additional procedure). | Yes ☐ | No☐ |
| I consent to being contacted after this study to ask if I would like to participate in follow-up research. | Yes ☐ | No☐ |

- - I want to participate in this research.

My name is (subject): .....................................

Signature: ........................... Date: __ / __ / __

I declare that I have fully informed this subject about the mentioned research.

If information becomes known during the research that could influence the consent of the subject, I will inform this subject in time.

Name of researcher (or their representative):.....................................

Signature:........................... Date: __ / __ / __

<if applicable>

Additional information was given by:

Name:......................................

Function:....................................

Signature:........................... Date: __ / __ / __

***The subject receives a complete information letter, together with a signed version of the consent form.***
