# Supplementary material for: Preoperative partial breast reirradiation and repeat breast-conserving surgery in patients with recurrent breast cancer: the prospective single-arm REPEAT trial – a study protocol
Source: BMJ Open. 2025 Jul 18;15(7):e096510. doi: 10.1136/bmjopen-2024-096510 (PMC12273098; doi:10.1136/bmjopen-2024-096510)
Supplement: Supplementary file 3 [file bmjopen-2024-096510.reviewer_comments.pdf]

PEER REVIEW HISTORY

BMJ Open publishes all reviews undertaken for accepted manuscripts. Reviewers are asked to complete a checklist review form and are provided with free text boxes to elaborate on their assessment. These free text comments are reproduced below.

ARTICLE DETAILS

Title (Provisional)

Preoperative partial breast reirradiation and repeat breast-conserving surgery in patients with recurrent breast cancer: the prospective single-arm REPEAT trial – a study protocol

Authors

Civil, Yasmin A.; Wurfbain, Lisca F.; Jonker, Lysanne W.; van der Sangen, Maurice; Oei, Arlene L.; Duvivier, Katya M.; Bijker, Nina; Meijnen, Philip; van Kesteren, Zdenko; Palacios, Miguel A.; Barbé, Ellis; Menke-van der Houven van Oordt, Willemien; Diepenhorst, Gwen; Thijssen, Victor L.J.L.; Slotman, Berend J.; Verhoeff, Joost J.C.; Schipper, Robert Jan; van den Bongard, Desirée

---

VERSION 1 - REVIEW

**Reviewer**	**1**
**Name**	**Kunkler, Ian**
**Affiliation**	**Western General Hospital, University Department of Clinical Oncology**
**Date**	**13-Feb-2025**
**COI**	**None**

Civil and colleagues present a clinically relevant protocol assessing the role of reirradiation with a single dose of preoperative partial breast irradiation in patients with small,unifocal, invasive local recurrence following initial breast conserving surgery and postoperative whole breast irradiation for invasive early breast cancer.

Specific points

Abstract page 6

line 63 English 'Advances'

line 76. Liquid biopsy. I am not aware of evidence to suggest that there is sufficient cDNA detectable by current methods for liquid biopsy in early breast cancer. This usually involves collection of a single volume of blood (perhaps 20-30 ml) to be collected for analysis. I am not sure this is justified. I do not have the Patient Information Sheet to know how the need

for this is explained. How many samples are proposed in what volume and at what frequency?

page 8

line 111 The authors need to define what they mean by breast conserving therapy.

This may include patients who undergo postoperative radiotherapy after breast conserving surgery with or without a boost dose of irradiation to the site of excision. Patient who have had a boost and develop a local recurrence in the boost area might be at risk of radiation necrosis if reirradiated to 20 Gy by PBI to the tumour and 15Gy to 2cm surrounding area. Are patients who received a boost dose as primary therapy eligible for the study or not?

The authors have not specified the dose-fractionation regimes for primary therapy that would be acceptable for eligibility for the study. These should be defined.

lines 128-130. The authors rightly point out that patients with a poor cosmetic result from breast conserving surgery and postoperative whole breast irradiation may be at risk of further adverse cosmetic outcome if re-irradiated. However I cannot see this as an exclusion criterion in Table 1.

page 9 line 145. What does 'based on grade 2 or higher toxicity. Are grades of toxicity including grade 1 to be reported?

line 148. The authors need to specify what biomarkers they propose to measure at a what frequency. Are this specified on the patient information sheet?

line 156. The authors specify 'single dose PBI followed by BCS

after three weeks.

What flexibility is allowable in the timing of surgery < or > 3 weeks?

page 10

line 160 I think this should read 'late radiation induced toxicity'

line 162 Specify what RT-associated response markers are to be measured.

Table 1

Are patients who have developed complications of primary BCS eg delayed wound healing eligible who may be at risk of adverse effects of re-irradiation?

Are patients with invasive breast cancer with an. introduction component eligible for the study?

page 12

line 189 What happens if the patient is intolerance of preoperative MRI (eg claustrophobia). Does the patient leave the study?

lines 189-190. Measurement of the pathological response after 2nd BCS is unlikely to informative give only 3 weeks been preoperative PBI and 2nd BCS.

line 190 I think the authors mean 'assessed' rather than 'scored'

Procedures

lines 205 and 206 and 208The authors do not specify the nature of the 'health professional' informing the patient about the study. It should be a radiation/clinical oncology consultant or senior trainee for a study of this nature.

page 13 Diagnostic work up

line 212 The authors need to justify why a PET scan is required for restating. It delivers significant radiation dose. Would breast imaging and ultrasound and bone scan plus chest radiograph suffice?

lines 215-216. What is the justification for additional tumour biopsies?

line 227-8 What account has been taken of the impact of previous whole breast irradiation +/-boost dose on normal tissue tolerance in selecting the 15 Gy and 20 Gy doses? These are doses that might be used for boost dose to site of excision in primary therapy. Is there any radiobiological basis for the selection of the preoperative PBI doses?

page 14 Radiotherapy treatment delivery

line 238 The authors need to be specific amount limit of no days less than or in excess of 14 days that permit eligibility for preop PBI after RT planning.

page 15

line 245 I cannot see the questionnaire appended. How long on average should it take a patient to complete and at what frequency?

Preoperative follow up

line 247 How many days < 21 or > 21 days are allowed?

line 250 The authors should specify what the blood samples are for

Breast conserving surgery

line 252 what are acceptable limits of timing of surgery (ie no of days +/- 21 days)?

page 16

lines 278 define what microscopic findings in the surgical specimen necessitating additional adjuvant systemic therapy.

page 17

lines 303-304 The authors specify submission to peer reviewed journals. Publication cannot be guaranteed.

page 18.

lines 314-315 It would be unusual to have only one member of a DMEC. Normally there are 3 to ensure range of views can be expressed.

Statistical analysis

lines 323-324 I think the unacceptable toxicity needs to be more tightly defined in terms of maximum number of patients experiencing > grade 2 toxicity. What the the stopping rules for the study? Would it not be better to test the regime on first 5 patients and only continue in 5 patient cohorts if toxicity up to the 21 patients.

What account will be taken in attrition to the study in determining the sample size?

line 334 How will the natural variability of the biomarkers be assessed?

page 19

line 351 English 'advances'

**Reviewer**	**2**
**Name**	**Matrai, Zoltan**
**Affiliation**	**Hamad Medical Corporation**
**Date**	**23-Apr-2025**
**COI**	**None**

Thank you for the invitation to review the manuscript titled Preoperative partial breast reirradiation and repeat breastconserving surgery in patients with recurrent breast cancer: the prospective single-arm REPEAT trial – a study protocol”

The aim and hypothesis of the study is with high interest in the oncoplastic era of breast oncology. Although the incidence of local recurrence is low after BCT, the need of safe secondary breast conserving surgery is there for many of the patients. Due to follow up protocols of breast cancer patients in most of the cases, local recurrence is diagnosed in a small size and early stage, making organ conservation technically possible. Although the carcinogenetic subcellular and cellular mechanism of a local and systemic recurrence is not much understood, the effect of possible reirradiation should be studied and evaluated in well controlled study circumstances.

I believe that the 5-year follow up is probably just a minimum, just think about the meta-analysis of the EBCTCG in 2005 Lancet, which reported that four prevented local recurrences at 5 years would avoid one breast cancer death in 15 years.

“Two studies on BCS with partial breast reirradiation using external beam 126 radiotherapy (RT) and brachytherapy reported 5-year OS rates of 95% (95% CI 85-98%) and 89% (95% 127 CI 86-93%), respectively (6, 11)” Yes, I agree.

I think, local control or disease free or recurrence free survival is more important to assess the effect or reirradiation, because overall survival is more related to systemic treatment effect even if some of the patients are already in stage IV.

“Second BCT might not be the preferred treatment for patients who 129 already had poor cosmetic outcome after initial BCT, as reirradiation could worsen cosmetic outcome 130 due to the risk of additional breast fibrosis. “ Agree completely, but please don’t forget that volume replacement surgeries with chest wall perforator flaps will have a measurable effect on higher breast conserving rates, because implant based postmastectomy breast reconstruction after RT is also associated with a higher rate of complications.

“A recent systematic review showed that grade 1 136 acute skin toxicity (0-34%), grade 1 seroma and grade 1 breast fibrosis (46-100%) were the most 137 frequently reported toxicities (12)” This point, the radiotherapy related early and late toxicities are of high interest, because most surgeons, by doing the follow ups of BCT patients, agree that in most of the radiotherapy studies too report that RT effect is up to grade 1, not more, while in clinical practice we all know well the frequent formation of fibrosis, pneumonitis, teleangiectasias, discoloration, invert pigmentation of NAC, capsular contractures which are not much reported and probably underestimated in RT studies. It highlights again the importance of long term follow up!

“Cosmetic outcome was good to excellent in 78 to 100% of the 138 patients.” I feel obligated to express my scepticism with regards to the 100%.

“The REPEAT trial is a Dutch multicenter, phase II, single-arm prospective study at the Amsterdam 155 University Medical Center and Catharina Hospital Eindhoven in the Netherlands” I think this study could work as a multicenter one only, due to the low number of events.

“Cosmetic outcome will be assessed 193 by the patients using the BREAST-Q questionnaire” Cosmetic PROMS but cosmetic objectives can better be measured by BCCT.core

DOI: 10.1016/j.cmpb.2015.11.010

“Additionally, cosmetic outcome 195 will be evaluated objectively using digital photographs analyzed by the BCCT.core software and 3D 196 Vectra XT® software”

I agree and this toll has to be prioritized and additional to PROMS.

I’m unable to find the surgical method of wide local excision in the protocol, whether the excision is done with open surgical cavity, traditional or oncoplastic technique, closed cavity techniques which would influence cosmetic outcomes in a significant way! That has to be standardized to be able to measure the RT effect.

VERSION 1 - AUTHOR RESPONSE

Reviewer: 1

Dr. Ian Kunkler, Western General Hospital

Comments to the Author:

Dear Dr. Kunkler,

Thank you very much for your kind words and valuable feedback. Please find below our point-by-point answers.

Abstract page 6

line 63 English 'Advances'

We adjusted this accordingly.

line 76. Liquid biopsy. I am not aware of evidence to suggest that there is sufficient cDNA detectable by current methods for liquid biopsy in early breast cancer. This usually involves collection of a single volume of blood (perhaps 20-30 ml) to be collected for analysis. I am not sure this is justified. I do not have the Patient Information Sheet to know how the need for this is explained. How many samples are proposed in what volume and at what frequency?

We have added the patient consent form as supplementary material. This form informs patients about the drawing of 52 mL blood twice. The study by Zhang et al. (2019) published in Clinical Cancer Research reported a ctDNA detection rate of 56% in patients with cT1 breast cancer in two gene panels analysed using DNA sequencing. The advances in sequencing techniques for ctDNA analyses, makes it possible to detect the low levels of ctDNA in early stage breast cancer. Especially, the tumor-informed approaches which requires prior sequencing of the tumor (Panet et al. 2024, npj breast cancer).

In the Patient Consent form we do not explain the ctDNA analyses since for now we are not able to provide an exact analysis method, as this evolves continuously. At this point in time

we therefore only take samples for storage. We will ask additional approval in the near future when there is a clear method/techique for the ctDNA analyses.

page 8

line 111 The authors need to define what they mean by breast conserving therapy.

The definition of breast-conserving therapy we use is surgery followed by radiotherapy as described in line 111.

This may include patients who undergo postoperative radiotherapy after breast conserving surgery with or without a boost dose of irradiation to the site of excision. Patient who have had a boost and develop a local recurrence in the boost area might be at risk of radiation necrosis if reirradiated to 20 Gy by PBI to the tumour and 15Gy to 2cm surrounding area. Are patients who received a boost dose as primary therapy eligible for the study or not?

We do not make a distinction between patients who received a boost and who did not, as we mainly focus on the clinical side effects of the primary radiotherapy treatment. Therefore, we do not mention anything about the boost.

The authors have not specified the dose-fractionation regimes for primary therapy that would be acceptable for eligibility for the study. These should be defined.

We deliberately chose not to specify the dose-fractionation regimens used in the primary therapy. Instead, we based our eligibility criteria on the observed toxicity resulting from the primary radiotherapy. Given that patients can respond quite differently to identical dose-fractionation schedules, we believe that the clinical outcome is a more meaningful and individualized measure. Therefore, patients who experienced toxicity greater than grade 2 following their initial radiotherapy were deemed ineligible for re-irradiation.

lines 128-130. The authors rightly point out that patients with a poor cosmetic result from breast conserving surgery and postoperative whole breast irradiation may be at risk of further adverse cosmetic outcome if re-irradiated. However I cannot see this as an exclusion criterion in Table 1.

Thank you, this is correct. We included this as an inclusion criterium in Table 1.

page 9 line 145. What does 'based on grade 2 or higher toxicity. Are grades of toxicity including grade 1 to be reported?

Indeed, grade 1 is also being reported. We further specified this in line 189.

line 148. The authors need to specify what biomarkers they propose to measure at a what frequency. Are this specified on the patient information sheet?

The biomarkers we want to evaluated are immune-activation and -suppressor markers. We also specified this in line 202.

The following information is specified in de Patient Information sheet: “Subsequently, six tubes of blood will be taken to conduct research on certain substances in the blood that can provide information about specific characteristics of the tumor and sensitivity to radiation, so-called marker substances.”

line 156. The authors specify 'single dose PBI followed by BCS after three weeks.

What flexibility is allowable in the timing of surgery < or > 3 weeks?

We added a more flexible time window of 1 week into lines 157,159, 252.

page 10

line 160 I think this should read 'late radiation induced toxicity'

We chose to use the term treatment-induced toxicity as one of the toxicities we report is surgery related. Therefore, we chose to use this more general term.

line 162 Specify what RT-associated response markers are to be measured.

We aim to measure immune stimulatory and suppressing T cells, such as CD4, CD8 and FOXP3 expressing T cells. We added this as well in the protocol (line 164).

Table 1

Are patients who have developed complications of primary BCS eg delayed wound healing eligible who may be at risk of adverse effects of re-irradiation?

This is a very valid point. In the inclusion criteria, we added that patients who experienced grade 2 or higher late toxicity will not be eligible for participation.

Are patients with invasive breast cancer with an. introduction component eligible for the study?

You probably mean DCIS component? Patients are indeed eligible according to the inclusion criteria, with a maximal size of the DCIS component of 2 cm.

page 12

line 189 What happens if the patient is intolerance of preoperative MRI (eg claustrophobia). Does the patient leave the study?

Unfortunately this can happen. Of course we will try to make the MRI as comfortable as possible. In case the patient still decides to quit participation, before receiving the study treatment, we will replace this participant.

lines 189-190. Measurement of the pathological response after 2nd BCS is unlikely to informative give only 3 weeks been preoperative PBI and 2nd BCS.

This is a very valid point. We do not expect much pathological response after this time interval. We are interested in semi-quantitative response assessment on MRI after this time interval. Although the complete response rate will be low, based on the ABLATIVE results we might expect some partial responses at three weeks after re-irradiation.

line 190 I think the authors mean 'assessed' rather than 'scored'

Thank you for this correction, we changed it (line 192).

Procedures

lines 205 and 206 and 208 The authors do not specify the nature of the 'health professional' informing the patient about the study. It should be a radiation/clinical oncology consultant or senior trainee for a study of this nature.

We specified the health care professionals in lines 208 and 211.

page 13 Diagnostic work up

line 212 The authors need to justify why a PET scan is required for restaging. It delivers significant radiation dose. Would breast imaging and ultrasound and bone scan plus chest radiograph suffice?

It is standard of care in the Netherlands to use PET scan to detect any regional and distant metastases. In recurrent cases, PET outperforms conventional imaging (Groheux 2022). However some hospitals only do dissemination research in case of suspicion of regional or distant metastasis. Breast imaging and ultrasound is standard of care during the diagnostic phase. The bone scan and chest radiograph are not routinely used. All patients are discussed in the multidisciplinary tumour board meeting and adequate imaging is advised. In the light of the study context we choose for PET scan in all the patients for staging.

lines 215-216. What is the justification for additional tumour biopsies?

The diagnostic biopsy is often entirely utilized for diagnostic evaluation. To enable tumor tissue analysis prior to radiotherapy, additional tissue is required. However, since study participation is not known in advance at the time of the initial biopsy, it is not feasible to collect extra tissue during that first procedure.

line 227-8 What account has been taken of the impact of previous whole breast irradiation +/-boost dose on normal tissue tolerance in selecting the 15 Gy and 20 Gy doses? These are doses that might be used for boost dose to site of excision in primary therapy. Is there any radiobiological basis for the selection of the preoperative PBI doses?

The previous dose of the primary radiotherapy is not taken into account. Instead we take into account the grade of late toxicity following radiotherapy. We think this might be more helpful in determining eligibility for re-PBI. The administration of a single 20 Gy dose equates to a 73.7 Gy dose when delivered in 2 Gy fractions (equivalent dose in 2 Gy fractions, a/b 4.7), leading to a 100% 5 year tumor control probability for cT1N0 tumors.16,17 A single 15 Gy dose corresponds to an equivalent dose in 2 Gy fractions of 44.1 Gy (a/b 4.7), aligning with the standard hypofractionated regimen comprising 16 fractions of 2.66 Gy utilized at our institution.

page 14 Radiotherapy treatment delivery

line 238 The authors need to be specific amount limit of no days less than or in excess of 14 days that permit eligibility for preop PBI after RT planning.

We specified this in line 242.

page 15

line 245 I cannot see the questionnaire appended. How long on average should it take a patient to complete and at what frequency?

We added this in line 249, and a reference to the supplementary material file with this questionnaire.

Preoperative follow up

line 247 How many days < 21 or > 21 days are allowed?

We added a time window in line 252.

line 250 The authors should specify what the blood samples are for

We added the reason for the blood samples in lines 255-256.

Breast conserving surgery

line 252 what are acceptable limits of timing of surgery (ie no of days +/- 21 days)?

We added a time window in line 258.

page 16

lines 278 define what microscopic findings in the surgical specimen necessitating additional adjuvant systemic therapy.

We added a specification of these findings in line 284-285.

page 17

lines 303-304 The authors specify submission to peer reviewed journals. Publication cannot be guaranteed.

This is a valid point. Of course we will try our best. Therefore, we added ‘submission’, because we will at least give this a good try.

page 18.

lines 314-315 It would be unusual to have only one member of a DMEC. Normally there are 3 to ensure range of views can be expressed.

The ethical committee itself consists of more than 10 people. However, the monitoring itself and evaluation if the trial adheres to all regulations is performed by one external person. This is standard in the Netherlands for all trials conducted.

Statistical analysis

lines 323-324 I think the unacceptable toxicity needs to be more tightly defined in terms of maximum number of patients experiencing > grade 2 toxicity. What this the stopping rules for the study? Would it not be better to test the regime on first 5 patients and only continue in 5 patient cohorts if toxicity up to the 21 patients.

Thank you for this valuable suggestion. We specified this further in line 332-333.

What account will be taken in attrition to the study in determining the sample size?

We added a percentage of 15% to the sample size, which made it go up to 25 to account for patient drop out.

line 334 How will the natural variability of the biomarkers be assessed?

Sorry for the unclear information, we specified these analyses in lines 344-345.

page 19

line 351 English 'advances'

We deleted this paragraph.

Reviewer: 2

Dr. Zoltan Matrai, Hamad Medical Corporation

Comments to the Author:

I believe that the 5-year follow up is probably just a minimum, just think about the meta-analysis of the EBCTCG in 2005 Lancet, which reported that four prevented local recurrences at 5 years would avoid one breast cancer death in 15 years.

Thank you for your comments and valuable input. Five years is indeed a short duration for this outcome. Since this is a feasibility study we wanted to keep the follow-up duration short. In the sequential study a longer follow-up will be definitely implemented.

“Two studies on BCS with partial breast reirradiation using external beam 126 radiotherapy (RT) and brachytherapy reported 5-year OS rates of 95% (95% CI 85-98%) and 89% (95% 127 CI 86-93%), respectively (6, 11)” Yes, I agree.

I think, local control or disease free or recurrence free survival is more important to assess the effect or reirradiation, because overall survival is more related to systemic treatment effect even if some of the patients are already in stage IV.

“Second BCT might not be the preferred treatment for patients who 129 already had poor cosmetic outcome after initial BCT, as reirradiation could worsen cosmetic outcome 130 due to the risk of additional breast fibrosis. “ Agree completely, but please don’t forget that volume replacement surgeries with chest wall perforator flaps will have a measurable effect on higher breast conserving rates, because implant based postmastectomy breast reconstruction after RT is also associated with a higher rate of complications.

This is a valid point. Preoperative PBI will also aid the use of oncoplastic techniques. Postoperative PBI after oncoplastic surgery often causes difficulties in identifying the clips due to distortion of the breast tissue, leading to irradiation of a larger breast volume. Preoperative PBI will solve this problem.

“A recent systematic review showed that grade 1 136 acute skin toxicity (0-34%), grade 1 seroma and grade 1 breast fibrosis (46-100%) were the most 137 frequently reported toxicities (12)” This point, the radiotherapy related early and late toxicities are of high interest, because most surgeons, by doing the follow ups of BCT patients, agree that in most of the radiotherapy studies too report that RT effect is up to grade 1, not more, while in clinical practice we all know well the frequent formation of fibrosis, pneumonitis, teleangiectasias, discoloration, invert pigmentation of NAC, capsular contractures which are not much reported and probably underestimated in RT studies. It highlights again the importance of long term follow up!

This is a very valid point. A longer follow-up will definitely be implemented in a sequential study when feasibility is proved.

“Cosmetic outcome was good to excellent in 78 to 100% of the 138 patients.” I feel obligated to express my scepticism with regards to the 100%.

This is very understandable and suggests indeed some detection bias in the study reporting 100% excellent cosmesis.

“The REPEAT trial is a Dutch multicenter, phase II, single-arm prospective study at the Amsterdam 155 University Medical Center and Catharina Hospital Eindhoven in the Netherlands” I think this study could work as a multicenter one only, due to the low number of events.

“Cosmetic outcome will be assessed 193 by the patients using the BREAST-Q questionnaire” Cosmetic PROMS but cosmetic objectives can better be measured by BCCT.core

DOI: 10.1016/j.cmpb.2015.11.010

“Additionally, cosmetic outcome 195 will be evaluated objectively using digital photographs analyzed by the BCCT.core software and 3D 196 Vectra XT® software”

I agree and this toll has to be prioritized and additional to PROMS.

We agree. Patient reported outcomes are the most important for evaluation of cosmetic results. However, to be complete we wanted to evaluate this in different ways.

I’m unable to find the surgical method of wide local excision in the protocol, whether the excision is done with open surgical cavity, traditional or oncoplastic technique, closed cavity techniques which would influence cosmetic outcomes in a significant way! That has to be standardized to be able to measure the RT effect.

The surgical techniques will be extensively described in the article that will be published. Standard surgery in the Netherlands is performed with full thickness closure. Oncoplastic surgical techniques are not often performed in these small tumors. Different surgical localization techniques used in different hospitals. We do not want to interfere in the general clinical practice. Therefore, surgeons are allowed to choose their own techniques, and these are always discussed in the multidisciplinary tumor boards.

VERSION 2 - REVIEW

Reviewer 1

Name Kunkler, Ian

Affiliation Western General Hospital, University Department of Clinical Oncology

Date 02-Jun-2025

COI

---

line 76 Liquid biopsy

I accept that liquid biopsy is covered under research on 'certain substances in the blood can provide information about specific characteristics of the tumor'

page 8 Definition of breast conserving therapy.

The authors do not know whether patients who have a tumour bed boost after breast conserving surgery and whole breast irradiation will be at greater risk of being re-irradiated. It would be safer to exclude patients who have a recurrence at the site of the original boost.

Dose fractionation regimes lines 128-130

It think it is a mistake not to define what are acceptable dose-fractionation regimes for inclusion. Such regimes are well defined internationally. By including non standard dose fractionation regimes, the authors may be providing endorsement of non standard regimes in their results

Patients with poor cosmetic results from primary breast conserving surgery and postoperative radiotherapy should be excluded from the trial on the basis that further irradiation may make their cosmetic outcome worse. Poor cosmetic results from primary breast conserving therapy is not currently an exclusion criterion.

page 9,line 145 point accepted

line 148 specification of biomarkers -accepted and noted in the PIS.

line 156 flexibility of time of surgery - amendment accepted

line 160 treatment-induced toxicity - point accepted

line 162 RT-associated response markers -amendment accepted

Table 1 I am not sure whether your grading system includes delayed wound healing. If it does not, delayed wound healing need to be specifically stated as an exclusion criterion.

page 12

Preoperative MRI - if patient is intolerant of preoperative MRI they should be excluded from the study

lines 189-190 pathological response after 2nd BCS -response to my comment accepted

line 192 correction accepted

lines 205,206,208 specification of health professional -amendment accepted

line 212 PET in diagnostic work up. Justification accepted

lines 215-216 Justification for additional tumour biopsies accepted

lines 227-8 Justification of dose - accepted

line 238 response accepted

line 245. Questionnaire reviewed and response accepted

line 247 time window -response accepted

line 250 specification of what blood samples are for. Response accepted

line 252 time window for surgery -response accepted

line 278 findings requiring additional adjuvant systemic therapy -response accepted

lines 303-4 submission to peer reviewing journals -response accepted

lines 314-5 DMEC administration of trial -response accepted

lines 323-324 statistical analysis -response accepted

Attrition to the study - response accepted

line 334 variability of the biomarkers -response accepted

VERSION 2 - AUTHOR RESPONSE

Reviewer: 1

Dr. Ian Kunkler, Western General Hospital

Comments to the Author:

page 8 Definition of breast conserving therapy.

The authors do not know whether patients who have a tumour bed boost after breast conserving surgery and whole breast irradiation will be at greater risk of being re-irradiated. It would be safer to exclude patients who have a recurrence at the site of the original boost.

We exclude patients who experienced grade 2 or higher toxicity. This includes patients who developed breast fibrosis or chest wall pain following a boost. However, patients who received a boost as part of their primary treatment but did not develop grade 2 or higher toxicity will be included in this trial. This decision has been approved by the medical ethics committee and was determined by consensus among radiation-oncologist from both participating hospitals. We believe this is a justified criterion, as excluding all patients based on having received a boost (regardless of toxicity) would unnecessarily limit our study population. We hope you can agree with this rationale.

Dose fractionation regimes lines 128-130

It think it is a mistake not to define what are acceptable dose-fractionation regimes for inclusion. Such regimes are well defined internationally. By including non standard dose fractionation regimes, the authors may be providing endorsement of non standard regimes in their results.

In the Netherlands, we have used and are still using the international defined dose-fractionation schedules.

This was standard conventional fractionation until 2010. From 2011 the Canadian schedule (16x2.66 Gy), and later on the START schedule (15x2.67 Gy).From 2020 the FAST-Forward schedule (5x5.2 Gy).

Regarding the boost, a sequential boost has been used until 2020 (i.e. 8x2 Gy). Sometimes a higher boost was indicated due to treatment according to the Young Boost Trial (i.e. 13x2 Gy).

From 2011, sequential boost (5-8x2Gy) or concomitant boost (21x2.66 Gy) has been used, depending on the radiotherapy center. From 2017, concomitant boost was used predominantly (20x2.67 Gy). Since 2023, we use the UK-boost schedule (15x3.2Gy).

Since the risk of breast fibrosis can also be increased by inhomogeneous plans e.g. in obese women, 3D RT (instead of static or volumetric IMRT), high RT boost volumes, we have decided not to include patients with breast fibrosis grade 2 or more.

Patients with poor cosmetic results from primary breast conserving surgery and postoperative radiotherapy should be excluded from the trial on the basis that further irradiation may make their cosmetic outcome worse. Poor cosmetic results from primary breast conserving therapy is not currently an exclusion criterion.

Sorry for the misunderstanding. We thought that fibrosis grade 2 or higher would be in line with poor cosmetic results. To be clear, we added this to the exclusion criteria.

Table 1 I am not sure whether your grading system includes delayed wound healing. If it does not, delayed wound healing need to be specifically stated as an exclusion criterion.

We included this as an exclusion criterion.

page 12

Preoperative MRI - if patient is intolerant of preoperative MRI they should be excluded from the study

Thank you for this comments. We added it as exclusion criterium.
